# Acute Seizure Susceptibility and Chronic Vascular Malformation in a Developmental Mouse Model of Sturge–Weber Syndrome

**DOI:** 10.3390/ijms27083519

**Published:** 2026-04-15

**Authors:** Nicholas Truver, Chase Solomon, Anne Comi

**Affiliations:** 1 Department of Neurology and Developmental Medicine, Hugo Moser Kennedy Krieger Research Institute, Baltimore, MD 21205, USA; 2 Department of Neurology, Johns Hopkins School of Medicine, Baltimore, MD 21205, USA; 3 Department of Pediatrics, Johns Hopkins School of Medicine, Baltimore, MD 21205, USA

**Keywords:** Sturge–Weber Syndrome (SWS), mouse model, Blood–Brain Barrier (BBB), Kainic Acid/Kainate (KA)-induced seizure, micro vessels, Immunohistochemistry (IHC)

## Abstract

Sturge–Weber Syndrome (SWS) is a rare neurovascular disorder caused by a somatic mosaic missense point mutation in *GNAQ,* resulting in a facial capillary malformation (port-wine birthmark) and abnormal blood vessels in the eye and brain. Symptoms include seizures, stroke-like episodes, and glaucoma. Acute seizures induced with low-dose kainate in a transgenic *GNAQ* R183Q mouse model of SWS assessed seizure susceptibility and the impact of seizures on cerebral vasculature. Mice expressing human *GNAQ* mutation experienced more severe seizures and greater seizure-induced mortality compared to littermate controls. Mutant mice had longer cortical microvessels, with larger diameters; the expression of tight junction proteins was reduced 2 days after seizures. Blood–brain barrier permeability was not different from controls after chronic gene expression, although vascular dilation persisted compared to controls. These data demonstrate increased seizure susceptibility in this somatic mosaic model of SWS, bidirectional interactions between seizure and vascular remodeling, and chronic persistence of vascular malformation.

## 1. Introduction

Sturge–Weber Syndrome (SWS) is a rare, congenital neurovascular disorder characterized by abnormal, enlarged blood vessels in the brain, skin, and eyes. Common symptoms of SWS include, but are not limited to, seizures, stroke-like episodes, cognitive and behavioral deficits, a facial port-wine birthmark, and glaucoma. Typical diagnosis of SWS involves a contrast-enhanced MRI of the brain showing leptomeningeal enhancement. There is currently no known cure; patients are generally treated individually based on symptoms with antiepileptic medications and low-dose aspirin. Epileptic seizures in people with SWS typically begin within the first 2 years of life [1,2,3]. Seizures present as focal seizures that can generalize and often include tonic–clonic movements. The early onset of seizures is often associated with more severe neurological outcomes, including cognitive decline and motor impairments [4]. Early epileptic seizures can be particularly difficult to treat and can be resistant to anti-seizure medications. Recently, evidence demonstrated that presymptomatic treatment (initiated prior to the onset of seizures) may improve outcomes, suggesting that SWS brain injury associated with seizures and impaired blood flow may be modifiable. Presymptomatic treatment with an anti-seizure medication and low-dose aspirin can delay or prevent seizures and improve SWS neuroscores at 2 years of age [3,5,6].

SWS results from a somatic mosaic missense mutation: 80–90% of SWS cases result from *GNAQ* R183Q [7]. This mutation (c.548G→A) results in impaired ability for protein Gαq to dissociate GTP; the inability to dissociate from GTP causes the constitutive activation of Gαq. Previous research has focused on the effects of hyperactive Gαq on its downstream pathways, primarily the Ras-Raf-MEK-ERK and mTOR pathways [8,9,10,11,12,13,14]. Importantly, the R183Q mutation in *GNAQ* is enriched in endothelial cells isolated from both brain and skin capillary malformations [15,16]. Tissue from these capillary malformations demonstrate significantly reduced levels of junction proteins such as zona occludens-1 and claudin-5 and increased vascular and blood–brain barrier (BBB) permeability [17].

The recently developed cerebrovascular mouse model for SWS (*tet*O-GNAQ*R183Q × Tie2*-rtTA/TRE-βGal*) produces mutant Gαq expression in endothelial cells. These mice demonstrate abnormally increased BBB permeability. At P15, mutant mice exhibit abnormal Evans blue staining of the cerebral cortex and irregular microvascular claudin-5 expression [18]. Brain endothelial cells, under normal conditions, exhibit low rates of transcytosis, which inhibit the transport of hydrophilic molecules between blood and the brain. Mouse models studying claudin and occludin proteins show their importance to tight junction integrity [19].

The studies presented here aimed to determine (1) the seizure susceptibility of these mutant mice and seizure-related mortality, and (2) the impact of seizures upon the expression of mutant Gαq, cerebrovascular structure, and the BBB. Because some mouse models of human epileptic disorders do not spontaneously develop seizures, kainate (KA), a kainate receptor agonist, is used to study the effects of seizures and to study seizure susceptibility [20]. We hypothesized that *GNAQ* R183Q mutant mice would demonstrate increased seizure susceptibility and mortality after low-dose KA, associated with abnormal vascular structure and BBB impairment. We also assessed BBB permeability and vascular structure in mutant transgenic mice at P42 to further delineate the age-related aspects of this model.

## 2. Results

### 2.1. Model with Kainate Injections at P15

**Figure 1:**
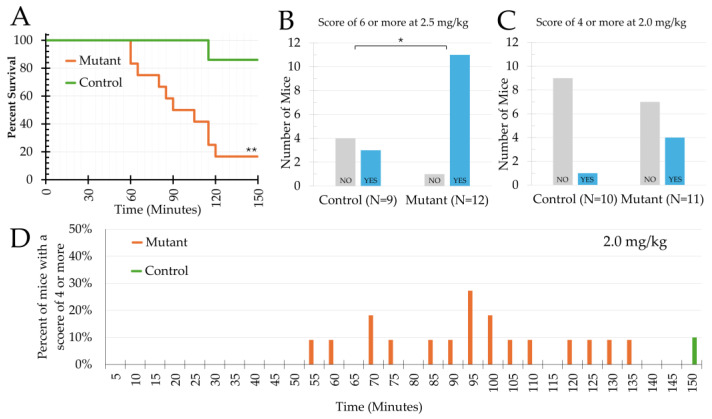
Seizure analyses based on Racine score observations at P15. (**A**) Kaplan–Meier survival curve for mice receiving the 2.5 mg/kg dose. (**B**) Number of mice that experienced a Racine score of 6 (tonic–clonic seizure) at the 2.5 mg/kg dose or (**C**) a Racine score of 4 at the 2.0 dose. (**D**) Percent of mice that experienced a score of 4 or more at each time point (note that the y-axis is in the range of 0–50%). * Indicates significance of *p* < 0.05; ** indicates significance of *p* < 0.005.

In pilot studies, doses of 3.0 mg/kg or greater were lethal in both SWS mutant and control mice; doses of 1.5 mg/kg and below elicited no response. For these studies, KA was injected at two doses into three transgenic litters each: 2.0 mg/kg and 2.5 mg/kg. The three litters that received the 2.0 mg/kg KA dose were made up of 21 mice, containing 10 control mice (5 males and 5 females) and 11 mutant mice (6 males and 5 females). While no mouse in the 2.0 mg/kg group died during the KA observation period, 2 mice died (1 mutant and 1 control, both female) before perfusion and were not used for further vessel analyses. The three litters in the 2.5 mg/kg KA dose were made up of 19 mice containing 7 control mice (3 males and 4 females) and 12 mutant mice (9 males and 3 females). Mutant mice had a significantly lower survival rate after 2.5 mg/kg KA administration compared to littermate controls (Figure 1; Kaplan–Meier, Log-Rank; χ^2^ = 8.148, df = 1, *p* < 0.005). A post hoc analysis of the sample sizes for acute mortality (α = 0.05, 0/7 controls died and 10/12 mutants died) achieved high power (1 − β) = 0.87. 

A significantly higher proportion of mutant mice had severe seizures (modified Racine score of 6) in the 2.5 mg/kg group compared to controls (11/12 vs. 3/9, *p* < 0.05, Fisher’s exact). Mutant mice in the 2.0 mg/kg group had more mice that experienced a score of 4 (not significantly 4/11 vs. 1/10; Figure 1). No score above 4 was observed in the 2.0 mg/kg group. A mixed-effects generalized linear model showed genotype as a significant predictor of the likelihood of severe seizures (Wald χ^2^(2, n = 630) = 25.69, *p* < 0.001; OR = 28.5, *p* < 0.05, 95% CI [1.6–490]). Sex was not a significant predictor of severe seizures (OR = 8.4, *p* = 0.15, 95% CI [0.47–153]). The mutant genotype was strongly predictive of severe seizures corresponding to an estimated increase in probability from 10% in controls to 76% in mutants. Mutant mice (n = 10; $\bar{x}$ = 5.53 cm; SEM = 0.07 cm) were significantly shorter than controls (n = 9; $\bar{x}$ = 5.80 cm; SEM = 0.08 cm) when measured at P17 after KA (*p* < 0.05); however, there was no significant difference in weight.

**Figure 2:**
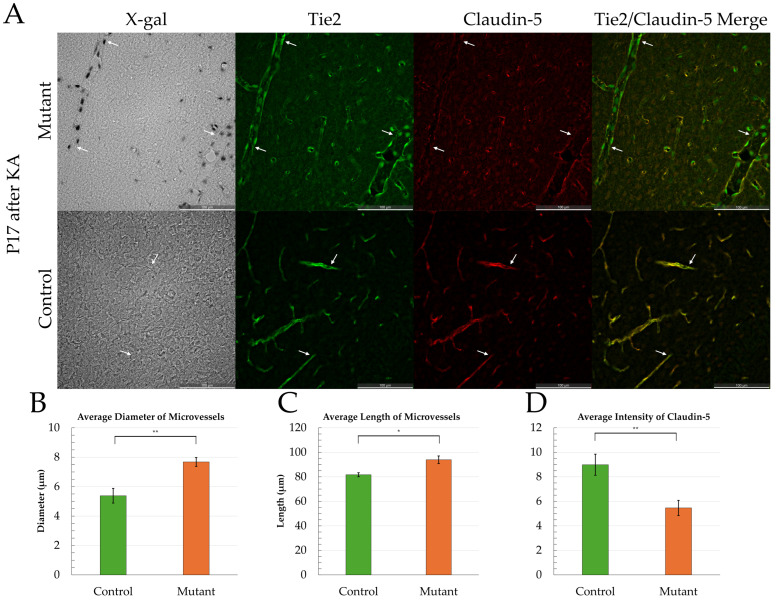
Examples of cortical tissue labeled with X-gal (signifying mutant gene expression), Tie2 (marking vasculature), and claudin-5 (BBB protein). (**A**) Example of microvessel sample area in mutant and control mice at P17. Comparison of (**B**) average diameter, (**C**) average length, and (**D**) average claudin-5 intensity between mutant and control tissue at P17. White arrows point to areas with less Claudin-5 staining (green areas in merged image). Error bars are +/− 2 SEM (* indicates significance of *p* < 0.05; ** indicates significance of *p* < 0.005). n = 19 mice; 9 controls (5 males, 4 females) and 10 mutants (6 males, 4 females). Four sample area images taken from each mouse (10 mutant and 9 control mice), a total of 402 microvessels analyzed (207 mutant and 195 control mice).

Because only 1 mutant mouse in the 2.5 mg/kg group survived KA injection, the following results of vessel morphology analyses only include mice from the 2.0 mg/kg KA group. There was no significant difference in the average number of vessels per sample area in mutant (n = 10) versus control mice (n = 9). The average mutant cortical microvessel length was ~15% longer than controls (mutant: n = 207 microvessels, $\bar{x}$ = 93.9 µm, SEM = 1.56 µm; control: n = 195 microvessels, $\bar{x}$ = 81.72 µm, SEM = 0.81 µm; *p* < 0.05; Figure 2). Average mutant vessel diameter was ~42% larger than cortical control microvessels (mutant: n = 207, $\bar{x}$ = 7.68 µm, SEM = 0.25 µm; control: n = 195, $\bar{x}$ = 5.38 µm, SEM = 0.15 µm; *p* < 0.005; Figure 2). Seizure score did not significantly correlate with average vessel length, diameter, or area in both mutant and control mice. Mutant microvessels had significantly less claudin-5 staining compared to controls (n = 207 microvessels, $\bar{x}$ = 6.32; n = 195 microvessels, $\bar{x}$ = 8.41; *p* < 0.005, Figure 2). In both mutant and control mice, average vessel diameter negatively correlates with claudin-5 intensity (r_s_(8) = −0.81, *p* < 0.05; r_s_(7) = −0.93, *p* < 0.05; respectively).

### 2.2. Chronic Model at P42

**Figure 3:**
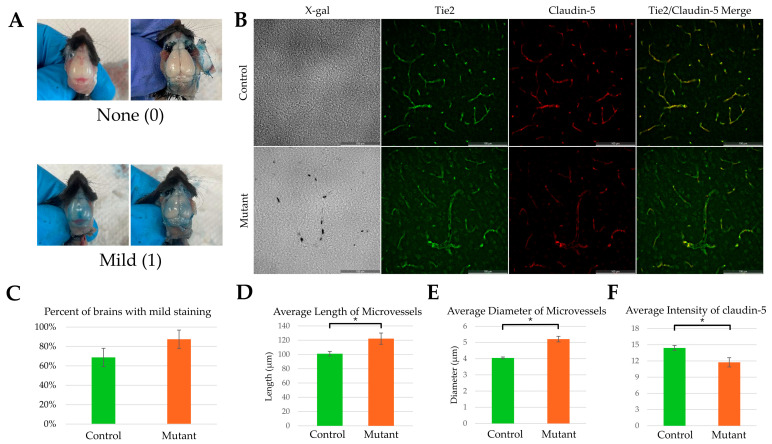
Evans blue at P42. (**A**) Examples of none and mild Evans blue staining with dura intact (left) and after the dura was removed (right) in a mouse brain. (**B**) Representative images of microvessel sample area in control and mutant tissue. (**C**) Percentage of mice with mild brain Evans blue staining; no significant difference in proportion of brains with mild Evans blue staining. Representative bar graphs of (**D**) average microvessel length, (**E**) average microvessel diameter, and (**F**) average intensity in control or mutant microvessels (* indicates significance of *p* < 0.05). Error bars are +/− 2 SEM. n = 24 mice: 16 control (5 males, 11 females) and 8 mutant (4 males, 4 females).

No grossly apparent abnormalities were noted in the skin or hair in mutant versus control mice. Weight and body length were significantly lower in mutant males compared to controls at P42 (weight: *p* < 0.05; body length: *p* < 0.05, Supplementary Table S1) but not in females. For the three litters of transgenic mice injected with Evans blue dye and immediately perfused at P42 (n = 24; 16 control mice, 5 males and 11 females; 8 mutant mice, 4 males, 4 females), no mice exhibited severe staining. There was no significant difference between the proportion of mutant and control mice that exhibited staining (control = 11/16, mutant = 7/8; *p* = 0.319; Figure 3).

Mutant mouse cortical brain tissue (n = 8 mice) had, on average, more microvessels per sample area compared to control (n = 16 mice) tissue (mutant = 29.0, SEM = 1.12, control = 24.6, SEM = 1.22; *p* < 0.05). Mutant microvessels demonstrated longer average vessel length compared to control microvessels (mutant n = 145 microvessels, $\bar{x}$ = 122.2 µm, SEM = 7.83; control n = 400 microvessels, $\bar{x}$ = 101.1 µm, SEM = 3.25; *p* < 0.05, Figure 3). Mutant microvessels had a significantly greater maximum diameter compared to control microvessels (mutant $\bar{x}$ = 7.61 µm, SEM = 0.24; control $\bar{x}$ = 5.87 µm, SEM = 0.09; *p* < 0.005) and an increased average diameter compared to controls (mutant $\bar{x}$ = 5.21 µm, SEM = 0.17 µm; control $\bar{x}$ = 4.05 µm, SEM = 0.06 µm; *p* < 0.005, Figure 3). Mutant microvessels had significantly less claudin-5 staining compared to controls (n = 240 microvessels, $\bar{x}$ = 11.74; n = 815 microvessels, $\bar{x}$ = 14.47; *p* < 0.005, Figure 3).

## 3. Discussion

**Figure 4:**
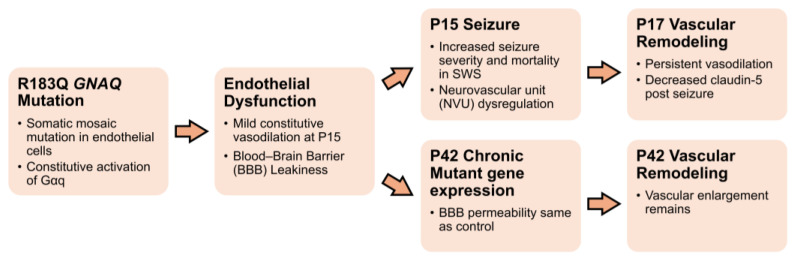
Conceptual overview of *GNAQ* mutation, BBB effects, impact of seizure, and age.

Seizures worsen cerebral blood flow in patients with SWS who have a vascular malformation in the brain, and result in venous stasis, ischemia, venous stroke, brain injury, and motor, visual, and neurodevelopmental impairments [21,22]. Understanding how seizures affect the neurovascular pathology of SWS is key to improving clinical management and quality of life for these patients. In this study, immature mice expressing the endothelial human mutant *GNAQ* R183Q gene experienced higher mortality during high doses of KA-induced seizures and more severe seizures during lower doses. In low-dose KA, there was a 7.6-fold increase in the probability of severe seizures in mutant mice compared to control mice, after the administration of low-dose kainate injections. In addition, mutant mice 2 days after KA-induced seizures demonstrated persistent vascular enlargement and decreased claudin-5 expression. These effects are briefly summarized in Figure 4. These novel results suggest that the endothelial brain somatic mutation in SWS increases seizure susceptibility, and the seizures may, in turn, persistently worsen neurovascular structure, function, and BBB impairment.

Two days after inducing a seizure with KA injection, mutant mice demonstrated a persistent abnormal dilation of cortical microvessels. In a typical young child, a seizure leads to increased cerebral blood flow and can cause transient focal enhancement in the leptomeningeal and cortical areas. This happens due to a temporary breakdown of the blood–brain barrier and dilation of the cerebrovascular system in the leptomeninges. In the setting of otherwise normal cerebral blood vessel structure and physiological responses, once seizure activity ceases, neurovascular recovery occurs rapidly, and little or no permanent neurologic or neurovascular injury is expected [23,24]. In our studies, mutant vessels were larger on average both before [18] and after seizures, suggesting a dysfunction of the neurovascular unit (NVU) before and after seizures. Qualitative inspection suggests more X-gal endothelial expression and larger vessels at P17 after seizure when compared to prior reported data at P15 [18]; while interesting, this observation requires future study and verification.

Claudin-5 is a crucial BBB protein in endothelial cells that form the BBB, along with other tight junction proteins, such as zonula occludens-1. Mutant mice showed ~25% lower cortical vascular claudin-5 staining after a KA-induced seizure compared to littermate controls. Mutant human Gαq activates downstream MAPK and NF-κB pathways and increases angiopoietin-2 (ANGPT2) expression, which destabilizes endothelial junctions and reduces baseline blood–brain barrier (BBB) integrity [12,25]. Seizures in the Tet-On SWS mouse model may exacerbate these pathways; however, further study is required. Given the observed reduction in claudin-5 expression and persistent vascular dilation in mutant mice post-seizure, therapeutic strategies targeting downstream pathways (e.g., MAPK and NF-κB) with targeted delivery to the BBB may offer potential benefits.

At P42 (without KA), no mutant mice demonstrated severe Evans blue staining, contrasting with the previous finding at P15 (also with no KA) [18]. As the BBB matures, it is possible that lower Gαq expression or other developmental (i.e., astrocytic or neuronal) changes occur that stabilize previously excessive BBB permeability. Interestingly, claudin-5 remains lower in mutant vessels, so the mechanism for the stabilization seen with Evans blue needs further study. Doxycycline, a tetracycline used to induce targeted mutant *GNAQ* R183Q gene expression, may also ameliorate vascular hyperpermeability in mouse endothelial cells [26,27,28]. Mutant male mice were lighter and shorter than male littermate controls at P42; the reason for this unexpected difference requires further study. Impaired growth may result from abnormal neuroendocrine or neurologic function, undetected seizure activity, or vascular impairments in other organ systems. It is noteworthy that patients with SWS have an increased risk of growth and other hormone deficiencies [29]; however, sex-related differences in growth are not known to occur. The precise mechanism and timing of BBB recovery require future elucidation. Repeated, longitudinal brain MRI studies assessing the BBB, gliosis, altered neuronal activity, and cerebral perfusion are needed in this model.

### Limitations

Scoring seizures based on a scale is semi-subjective. Studies of seizures in animal models often use electrodes and other EEG devices to accurately capture electrical activity more directly; however, there are technical challenges in mouse pups, including the fragility and smaller size of the skull, and increased background signal, which result in excessive mortality and severe artifacts that greatly limit EEG studies [30,31]. Therefore, we used the widely accepted Racine scale [18,20,32]. Seizure in SWS cases is often refractory and can occur repeatedly; therefore, data collection from repeated KA-induced seizures is needed to describe the chronic effects of seizure on microvasculature. Additionally, because three litters were used for each comparison, litter-dependent effects are possible, although this approach is consistent with prior genetic mouse studies [33,34].

Assessing sex-related differences in mouse models of seizure is complicated by strain-specific hormonal differences [35,36]. However, immature mouse models have shown sex-related differences in responses to seizures [37,38,39] and in response to ischemia [40,41,42]. Generally, in humans, males have a slightly higher incidence of seizure [43,44,45]; however, hormonal differences play a significant role in the onset and outcome of seizures as well [46,47,48]. Seizure severity and brain injury due to hypoxic ischemia may be impacted by hormones like estrogens and androgens or prohormones like progestogens [49,50,51]. In SWS patients, there is no difference in seizure onset; however, males are more likely to have stroke-like episodes [52]. Our results in this genetic mouse model of SWS suggest sex-related impacts upon later weights; studies that more specifically explore sex-related vascular remodeling after seizure in SWS are needed.

Future research, potentially including longitudinal study designs, electrophysiological techniques in adult mice, and chemical or gene editing modulation of the mutant protein, would be needed to directly determine the relationship between seizures and alterations in microvasculature in these mutant mice. Seizures are often triggered by illness and fever in infants with SWS; future studies could model this aspect with lipopolysaccharide (LPS) and/or elevated body temperature. In vivo molecular interactions are exceedingly complex, limiting our ability to understand cause and effect relationships between proteins and pathways; future in vitro studies of endothelial cells expressing the *GNAQ* R183Q mutation would be informative.

## 4. Materials and Methods

Protocol was approved by Johns Hopkins University Animal Care and Use Committee (Protocol number MO25K82, approved March 2025) and followed required local and NIH requirements. Animals were checked daily by animal facility staff and at least twice a week by study staff. Any animal demonstrating distress, including weight loss, hair loss, hunched position, other wounds, or concerns were evaluated and, if needed, euthanized. None of the animals required euthanasia.

### 4.1. Mouse Colony Management

All mice are housed in a central rodent animal facility at Johns Hopkins with normal housing conditions (12 h light/dark cycle, 20–22.2 °C room). Either a trained research assistant or the Research and Animal Resources staff at Johns Hopkins checked the mice daily. *tet*O-GNAQ*R183Q mice were backcrossed for at least 3 generations, then crossed with Tie2-*rtTA/TRE-βGal* mice from Jackson labs, which resulted in transgenic litters with 50% “control” mice (*tet*O-GNAQ*R183Q) and 50% “mutant” mice (*tet*O-GNAQ*R183Q × Tie2-*rtTA/TRE-βGal*). Note that control mice carry the human *GNAQ* mutation but cannot express it, whereas mutant mice have the human *GNAQ* mutation, which can be expressed in the presence of doxycycline. Each experimental group in this study consisted of multiple transgenic litters (i.e., two/three litters can make up one experimental group).

#### 4.1.1. Doxycycline Administration

Doxycycline was provided to all experimental transgenic litters through food pellets in the dam’s feed, initiated by E7, and was provided continuously, including post-weaning, until euthanasia and/or perfusion (Supplementary Figure S2). Doxycycline feed with a concentration of 2000 mg/kg was purchased from Envigo (Indianapolis, IN, USA, Cat. No. TD.09633). Doxycycline feed was changed weekly on Friday. Because the expression of the mutant gene has previously been seen with this doxycycline concentration in this model [18], the serum levels and amount of doxycycline intake were not measured in line with other Tet-On/Tet-Off models [53,54].

#### 4.1.2. Confirmation of Genotype

Mice tails were clipped at P14, kept at 4 °C until after the seizure scoring, and then processed for genotyping within 1 week after seizure scoring. Tail clippings were genotyped using the following primer sets, which correspond to the two separate portions of the Tet-On construct required for doxycycline expression of mutant Gαq in endothelial cells:

*tet*O-GNAQ*R183Q primers (positive *GNAQ* result shows a band at 360 bp):

Forward: TGAAGATGTTCGTGGACCTGAACCC.

Reverse: TTAAAGGCATTCCACCACTGCTCCC.

*Tie2-rtTA/TRE-βGal* primers (positive Tie2 result shows a band at both 200 bp and 450 bp; negative Tie2 result shows a band only at 200 bp):

Forward: CGCTGTGGGGCATTTTACTTTAG.

Reverse: CATGTCCAGATCGAAATCGTC.

Internal Positive Control Forward: CAAATGTTGCTTGTCTGGTG.

Internal Positive Control Reverse: GTCAGTCGAGTGCACAGTTT.

Primers were obtained from Integrated DNA Technologies (IDT, Madison, WI, USA). PCR of amplification of GNAQ was performed with an initial denaturation at 94 °C for 2 min, followed by 35 cycles of 94 °C, 60 °C, and 72 °C with a final extension at 72 °C for 5 min before holding at 10 °C. PCR of Tie2 was completed with an initial denaturation at 94 °C for 2 min, followed by 10 cycles at 94 °C, 65 °C, and 68 °C. An additional 28 cycles were performed at 94 °C, 60 °C, then 72 °C, with a final extension at 72 °C before holding at 10 °C. After running isolated DNA through PCR reactions using the primers listed above, as previously described [18], the products were electrophoresed at 80 V in a 1% agarose gel for 1 h. Gels were imaged using the GelDoc XR+ Imaging System from BioRad (Hercules, CA, USA) with Quantity One 4.6.8 software (ChemiDoc XRS setting).

#### 4.1.3. Mouse Numbers, Mortality, Weight, and Body Length

A total of 40 mice from 6 litters underwent kainate-induced seizures (Supplementary Figure S2). A total of 3 litters accounting for 21 mice were administered kainate i.p. at the 2.0 mg/kg dose. A total of 3 litters accounting for 19 mice were administered kainate i.p. at the 2.5 mg/kg dose. A total of 1 control and 1 mutant mouse died in the time between seizure analyses and perfusion in the 2.0 mg/kg group. A total of 24 mice from 3 litters were injected with Evans blue prior to perfusion. Sample sizes were based on our previous paper in this model, and our prior publications in another immature mouse model of stroke and seizures [18,31]. Mortality was assessed for all transgenic and maintenance mouse litters by calculating the percent of pups dead before P21 and percent of pups dead between P21 and P41. Body weight (grams) and length (centimeters) were measured prior to perfusion. Body length measurements were taken after anesthesia and before perfusion; a ruler was used to measure rump to nose body length.

### 4.2. Seizure Induction

A subset of mice was given kainic acid (KA; K0250-50MG from Millipore Sigma, Burlington, MA, USA) intraperitoneally (i.p.) at P15; two doses were chosen based on pilot study findings: 2.0 mg/kg and 2.5 mg/kg. Doses of kainate higher than 3.0 mg/kg resulted in mortality in both mutant and control mice, while doses lower than 2.0 mg/kg resulted in no seizure activity. KA was dissolved in sterile saline and injected into mice with 27G U-100 syringes at 1% of body weight (e.g., 60 µL for a 6 g mouse injection volume was 1% of body weight; volumes ranged from 50 to 70 µL). After injection, mice were placed in a temperature-controlled environment (~35 °C) and continuously monitored for 150 min. Every 5 min for 150 min, mice seizure activity was rated and noted in accordance to the modified Racine scale (Table 1; as previously published [30,31]). Mice tails were not genotyped until after seizure analyses to ensure that investigators were blinded to genotype when rating. Tails were kept at 4 °C until processed as described above. After analyses, mice were returned to their respective dams. At 48 h after seizure induction, mice were perfused. The weight of each mouse was noted at the time of seizure induction and at the time of perfusion. Because acute seizure mortality is an important outcome in SWS, mice were not treated prior to seizure or death; however, to mitigate suffering, if mice were still severely seizing at the end of the observational period, they were euthanized. No animals required euthanasia.

### 4.3. Perfusion, Brain Extraction, and Tissue Processing

Mice were sacrificed/perfused, either two days after seizure induction (at P17) or at P42 with Evans blue (Supplementary Figure S2). Mice were first anesthetized with isoflurane and confirmed with a toe pinch before being perfused with 1× PBS for 5 min, followed by 4% paraformaldehyde (PFA) with 0.5% glutaraldehyde for 6 min. The perfusion rate was 3 mL/min. In a subset of mice, 75 µL of Evans blue dye was perfused through the left ventricle before perfusion with PBS and PFA. Once the right atrium was clipped shortly after starting the perfusion pump, Evans blue was clearly seen going into the heart as well as the paws and chin/snout region of the mouse. Evans blue was followed by perfusion with 1× PBS and then PFA circulation for 5 min.

Following perfusion, brains were extracted and post-fixed in 4% PFA with 0.5% glutaraldehyde for 45 min, followed by 15% sucrose for 24 h, then 30% sucrose for 24 h. Brains were then flash frozen using dry ice and stored at −80 °C. A cryostat (MICROM HM 500 M) was used to section all brains at −22 °C into coronal, 20 µm sections. All images obtained were from anatomically matched areas in the retrosplenial cortex of coronal sections as described previously in [18]. Coronal, 20 µm sections were used to stain and visualize branching microvessels in the mouse pup brain sections (which are fragile), as has been widely established and published [55,56]. Sections were mounted to slides with PBS and stored at −80 °C until staining or IHC was done.

### 4.4. Immunohistochemistry (IHC) with Fluorescence

All tissue was first stained with X-gal using the β-Galactosidase Reporter Gene Staining Kit (Sigma-Aldrich, Saint Louis, MO, USA, #GALS) to detect LacZ-mediated mutant gene expression. Blocking done for 1 h at room temperature (RT) with a solution of 3% Normal Goat Serum (NGS) and 0.3% TritonX-100 in PBS. X-gal staining prior to IHC was used to spatially evaluate gene transcription in tissue [57,58] and was successfully employed in our prior manuscript with this model [18]. Tissue sections were then incubated for 2 h at RT with primary antibodies to target the following: Tie2 (1:100; Invitrogen, Rockford, IL, USA, #14-5987-82) and claudin-5 (1:100; Invitrogen, Waltham, MA, USA, #34-1600). After primary antibody incubation, tissue sections were incubated for 1 hr at RT with the following fluorescent tags: Alexa Fluor 594 (1:1000; Alexa Fluor 594-Goat-Anti-Rabbit, Invitrogen, #A-11037), Alexa Fluor 647 (1:1000; Alexa Fluor 647-Goat-anti-Rat, Invitrogen, #A48265), and finally DAPI (ProLong Gold Antifade Reagent with DAPI, Cell Signaling, Danvers, MA, USA, #8961S).

Primary antibodies were tested with negative controls in pilot studies to confirm specificity. Antigen retrieval is not required to expose these epitopes (Tie2, claudin-5) for the IHC performed in these perfused immature mouse brains; antigen retrieval is primarily required for paraffin-embedded tissue [59], which was not the case here.

### 4.5. Microvessel Morphology, Claudin-5, and X-Gal Analyses

For each animal, 4 non-overlapping images were obtained at 400× of cortical microvessels in anatomically matched areas of the retrosplenial cortex at the level of the anterior hippocampus as previously described [18]. Because this area is considered a “hotspot” of microvessels, there are fewer fields [60]. Each image contained a channel for Tie2, DAPI, X-gal, and claudin-5. All further histological image processing and image analyses were done blinded to genotype; randomly labeled file names were assigned to images by a separate investigator. The TIE2 channel images were processed in ImageJ (version 1.54p) to identify microvessels, first using the Yen auto-threshold adjustment and then the Mexican Hat Filter plugin. After that, any signal under 30 µm in length was removed in Photoshop (per AngioTool guidelines [61]), and holes in vessels were filled in; these final black and white masks were then analyzed for morphological characteristics such as vessel length, diameter, and branching in AngioTool and Fiji with ImageJ and saved as microvessel masks (Supplementary Figure S1).

To analyze claudin-5, Fiji/ImageJ was used to overlay microvessel masks (as described above) on the claudin-5 channel. Each microvessel was selected as an individual region of interest with the “analyze particles” tool, and the average intensity of claudin-5 was measured. Then, by selecting and combining all vessel regions of interest, an inverse selection was made to measure the average intensity of the background (Figure S1). The intensity of the background was subtracted from each vessel. This process was automated with a custom macro.

Maximum microvessel diameter was calculated by a blinded investigator using the straight-line measurement tool in ImageJ by drawing a line across the thickest portion of each microvessel. Average microvessel diameter for each microvessel was calculated using the macro VasoMetrics in Fiji with ImageJ. See McDowell et al. 2021 [62] for a description of how VasoMetrics is used to accurately measure vascular diameter.

AngioTool determined the total area of microvessels (µm^2^) and the vascular density, which is measured by dividing the total area of microvessels by the explant area (the area in which the highlighted microvessels occupy). AngioTool also calculates the number of end points of all microvessels within an image. The number of end points is directly proportional to the extent of microvascular branching; a higher total end points value indicates that, for a given image, the microvessels have more branches. See Zudaire et al. 2011 [61] for a comprehensive guide on AngioTool and its main functions and outputs.

The “analyze particles” tool in Fiji with ImageJ counted the labeling of X-gal in mutant tissue. This tool calculates the number of ellipses in an image after thresholding. Any ellipse smaller than 2 µm^2^ was deemed an artifact and thus not counted towards the total. For all morphological assessments of microvessels in mutant tissue, only microvessels with X-gal labeling would be considered for analyses. All microvessels, regardless of the presence of X-gal, were still counted in mutant tissue and compared to the number of microvessels present in control tissue. See Supplementary Figure S1 for how images of cortical microvessels were processed and computed in ImageJ (version 1.54p), Photoshop (2024), and AngioTool.

### 4.6. Statistical Analysis

All statistical analyses were carried out in SPSS (IBM SPSS Statistics for Windows, Version 27.0., Armonk, NY, USA). Levene’s test of equality of variance were done for t-test. Independent samples t-tests were used to compare weight, body length, and characteristics of microvessel vasculature in perfused transgenic mice. Mortality rates for mice were calculated using Fisher’s exact p-value. A mixed-effects generalized linear model (GLMM) with ordinal distribution and logit link function was fitted to model the probability of severe seizures. Genotype and sex were included as fixed effects, and standard errors were clustered by litter to account for within-litter dependence. Racine scores (assessed at 30 timepoints per animal) were treated as repeated observations within the GLMM; time was not included as an explicit predictor. Probability of mutant mice was calculated as the odds of control mice having a severe seizure (Racine score > 3 during the scoring period) multiplied by the estimated odds ratio (OR) from GLMM. A Chi-square test was also used to calculate the proportion of brains with mild Evans blue staining, as well as for the presence of seizure activity in our KA injection studies. Reported p-values reflect the Bonferroni correction for multiple comparisons of weights and microvessel analyses. A Kaplan–Meier Log-Rank test was used to calculate significant differences in survival during seizure analyses at P15. Analyses of trends for differences (in genotype versus controls) by sex were completed, and although no differences were identified when comparing seizure and microvascular data, differences in weights were seen at P42.

## 5. Conclusions

The SWS mouse model expressing the R183Q *GNAQ* mutation reveals complex developmental and neuropathological features that evolve with age and mirror several clinical aspects noted in patients with SWS. At P15, increased susceptibility and mortality to KA-induced seizures highlight the vulnerability of the immature brain in SWS cases. Persistent vasodilation and altered BBB proteins suggest dynamic interactions between seizure activity and vascular signaling pathways within the NVU. By P42, mutant mice exhibit growth impairments and altered vascular morphology yet show the resolution of early blood–brain barrier (BBB) defects. These findings underscore the importance of temporal analyses in understanding SWS pathophysiology and point to future directions exploring BBB recovery, and the long-term impact of seizure-induced vascular changes. These data expand the translational importance of this SWS mouse brain model, which can now be utilized preclinically to assess novel treatments for seizure susceptibility and mortality, devastating clinical aspects of SWS.

## Figures and Tables

**Table 1:** Modified Racine scale.

0	Normal Activity
1	Immobility
2	A period of forelimb and/or tail extension, giving the appearance of rigid posture
3	Automatisms such as circling or head bobbing/deviations
4	Forelimb clonus (sustained muscle retraction) and “popping” (myoclonic seizures)
5	Tonic seizures and falling over (tonic = sustained extension of muscles)
6	More severe, tonic–clonic, seizures (full body convulsions)
7	Death

## Data Availability

The original contributions presented in this study are included in the article/Supplementary Materials. Further inquiries can be directed to the corresponding authors.

## Supplementary Materials

The following supporting information can be downloaded at: https://www.mdpi.com/article/10.3390/ijms27083519/s1.
