# Supplementary material for: Acute Seizure Susceptibility and Chronic Vascular Malformation in a Developmental Mouse Model of Sturge–Weber Syndrome
Source: Int J Mol Sci. 2026 Apr 15;27(8):3519. doi: 10.3390/ijms27083519 (PMC13115601; doi:10.3390/ijms27083519)
Supplement: Supplementary file 1 [file ijms-27-03519-s001.zip › ijms-4125312-supplementary.pdf]

Supplementary Materials

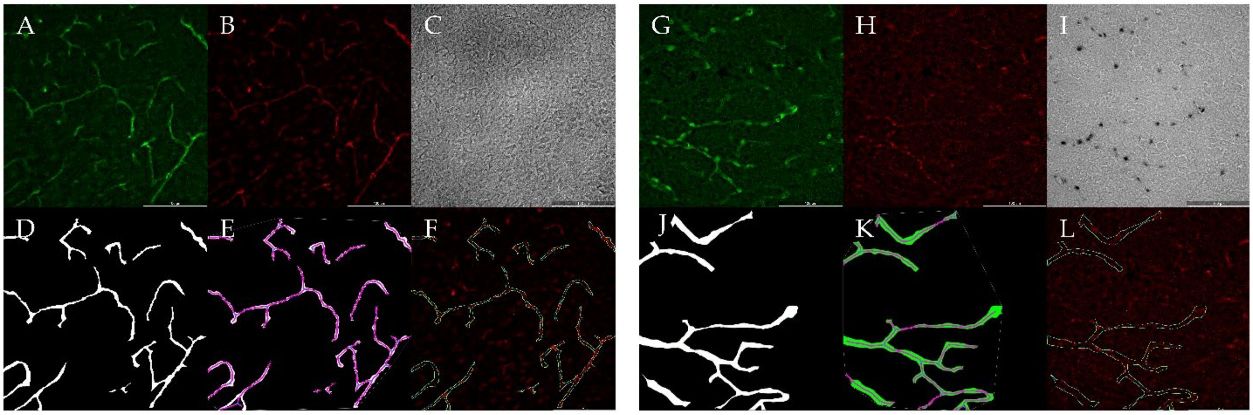

Supplementary Figure S1. Image quantification of microvessel morphology and claudin-5 protein expression. A-F are examples from control tissue, G-L are examples from mutant tissue. Images of Tie2 channel (A, G) and the claudin-5 channel (B, H) shown. A vessel mask was created from the Tie2 channel (D, J) which is then used for the microvessel morphology analysis with Angiotool (E, K). The vessel mask is then also used in an ImageJ macro to calculate the claudin-5 protein expression in the vessels after subtracting background (F, L).

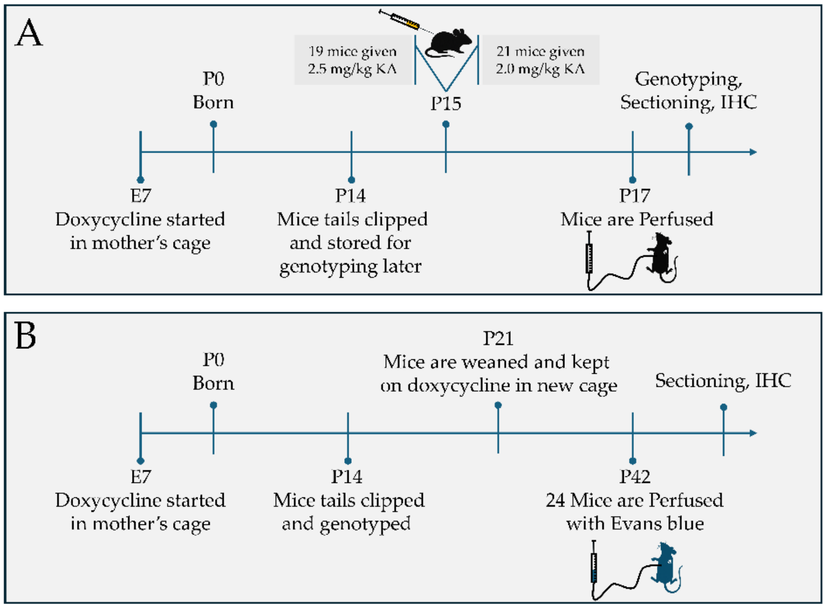

Supplementary Figure S2. Experimental Timelines. Timeline of events for mice (A) in the acute seizure study and for other mice (B) in the chronic gene expression study (B).

Supplemental Table S1. Average Weights and Body Lengths at perfusion (P42).
* Indicates significance at $p < 0.05$

87 total mice	# of mice	Weight (g)	Body length (cm)
Total Males	41		
Control	21	* 20.58	* 8.11
Mutant	20	19.28	7.90
Total Females	47		
Control	26	16.33	7.58
Mutant	20	16.30	7.61
